# Preparation of the Heterogeneous Saponified Poly(Vinyl Alcohol)/Poly(Methyl Methacrylate–Methallyl Alcohol) Blend Film

**DOI:** 10.3390/ma15072439

**Published:** 2022-03-25

**Authors:** Seong Baek Yang, Dae Won Jeong, Jungeon Lee, Sabina Yeasmin, Chang-Kil Kim, Jeong Hyun Yeum

**Affiliations:** 1 Department of Biofibers and Biomaterials Science, Kyungpook National University, Daegu 41566, Korea; ysb@knu.ac.kr (S.B.Y.); wjdeodnjs@gmail.com (D.W.J.); dlwjddjs2@gmail.com (J.L.); yeasminsabina44@yahoo.com (S.Y.); 2 Department of Horticultural Science, Kyungpook National University, Daegu 41566, Korea

**Keywords:** poly(vinyl acetate), poly(vinyl alcohol), poly(methyl methacrylate), solution casting, saponified film

## Abstract

For the first time, poly(vinyl alcohol) (PVA)/poly(methyl methacrylate–methallyl alcohol) (P(MMA-MAA)) (9:1, 7:3, 5:5) blend films were made simultaneously using the saponification method in a heterogeneous medium from poly(vinyl acetate) (PVAc)/poly(methyl methacrylate) (PMMA) (9:1, 7:3, 5:5) blend films, respectively. The surface morphology and characteristics of the films were investigated using optical microscopy (OM), atomic force microscopy (AFM), X-ray diffractometer (XRD), Fourier transform infrared (FTIR) spectroscopy, thermogravimetric analysis (TGA), and differential scanning calorimetry (DSC). Moreover, the effect of the PVAc content on the degree of saponification (DS) of the PVAc/PMMA films were evaluated and revealed that the obtained DS value increased with the increase in PVAc content in the PVAc/PMMA blend films. According to the OM results, the saponified films demonstrated increased surface roughness compared with the unsaponified films. The AFM images revealed morphological variation among the saponified PVAc/PMMA blend films with different mass ratios of 9:1, 7:3, and 5:5. According to the DSC and TGA results, all blend film types exhibited higher thermal property after the saponification treatment. The XRD and FTIR results confirmed the conversion of the PVAc/PMMA into PVA/P(MMA-MAA) films. Thus, our present work may give a new idea for making blend film as promising medical material with significant surface properties based on hydrophilic/hydrophobic strategy.

## 1. Introduction

The poly(vinyl acetate) (PVAc) is a thermoplastic polymer produced from the polymerization of the vinyl acetate amorphous polymer [1]. PVAc has been extensively studied in the literature owing to its outstanding optical properties, biocompatibility, slight foreign-body reactions in vivo [2], and good adhesion and coating effects [3,4,5]. The main application area of the PVAc are wood processing, civil engineering, packaging, and binding industry adhesives and coatings, construction, textile, paper, leather, biomedical, and other fields [6]. On the other hand, depending on its simple preparation, harmless, noncarcinogenic, eco-friendly and bio-adhesive nature, exceptional chemical resistance, and physical characteristics, poly(vinyl alcohol) (PVA), which is a water-soluble polyhydroxy polymer, has attracted great interest because of its potential applications [3,7,8]. It presents beneficial thermo-mechanical characteristics, mechanical strength and flexibility, and thermal stability along with good optical and physical characteristics that are essential for packaging application [9,10]. Besides, PVA is accepted by the food and drug administration as an unintended food additive for flexible food packaging application [11]. PVA, as exceptional biomaterials, can replicate natural tissues, lining for false organs, contact lenses, and drug release [3,7,8]. Although PVA possesses good mechanical characteristics under dry conditions, its high hydrophilicity limits its applications. Poly(methyl methacrylate) (PMMA), which is obtained from the polymerization of methyl methacrylate (MMA), is an important polymeric material with high light transmittance, is transparent, and demonstrates opposition to chemical and weather corrosion. These superior PMMA properties allow its practical application in a broad area such as in coating, outdoor electrical applications, and optical fiber [4,5,12]. Moreover, it has similar refractive index to that of the optically clear adhesive (OCA) usually used in the front of cell phone displays [13,14,15].

Several research studies have reported on converting PVAc to PVA and the obtained saponified PVA showed improved physical properties compared to PVA prepared using PVA solution [16]. For example, LEE et al. developed a method for the preparation of PVA beads (up to 1.5 mm diameter) with a core shell structure through a saponification of PVAc beads prepared by suspension polymerization followed by crosslinking of the PVA core and shell stepwise with glutaraldehyde and the resulting composite showed different outer shells and inner cores depending on the degree of crosslinking. Moreover, they showed the controlled saponification by keeping the methanol concentration below 10% to slow the reaction penetration as high concentrations of methanol induces rapid saponification of PVAc [17].

In other studies, it was shown that the saponification method was used in a useful way to prepare monodisperse PVA microspheres using salts such as NaSO_4_, which is helpful in minimizing the dissolution of PVA skin [18]. The effects of MMT on both the polymerization rate and the saponification rate of PVAc were investigated by Jung et al. who found that the polymerization rate decreased with increased MMT [19]. In our recent research, we have extended much effort to PVAc nanomaterial saponification under a heterogeneous condition and developed a novel technique as well as successfully produced several PVA-based fibers and films. In contrast to general PVA films, our heterogeneously saponified film demonstrated extraordinary black globules with distinct sizes in the film constitution [20], and the fiber exhibited extraordinary wrinkled and wound features [21].

Currently, polymer blending is the most practical technique for developing or changing the physicochemical characteristics of polymer materials [3]. The miscibility of the ingredients illustrates a significant characteristic of the polymer blend because it influences the mechanical characteristics, morphological properties, permeability, and degradation time [3,22]. Several studies on the miscibility of multi-component polymer techniques have been reported. Because most polymers are incompatible, we need to design the polymer structure and blending method according to the purpose of obtaining desirable polymer blends [23].

Because of their hydrophilicity, some polymer films have limited applications. Therefore, blending them with a hydrophobic polymer would be a good alternative to address this issue, and few reports on this subject are available. Zhu et al. presented a unique method of making a sequence of PVA/poly(butyl acrylate-co-methyl meth-acrylate) [PVA/P(BA-co-MMA)] blend films with different P(BA-co-MMA) concentrations using the solution-casting method [24]. Another report demonstrated that the introduction of a polyacrylonitrile part improved the hydrophobicity of the polymer blend films [25]. The preparation of polypeptide-based film blending with poly(acrylic acid) is a good example of a hydrophilic–hydrophobic blend film production [26].

In the present study, we also attempted to produce a hydrophilic PVA blend film with hydrophobic–hydrophilic P(MMA-MAA). As PVA has some fundamental deficiencies, for example, poor moisture barrier, water resistance properties, and thermal stabilities, which greatly prevent its applications in food packaging so the incorporation of poly(methyl methacrylate) (PMMA) can overcome the drawbacks. More significantly, PMMA has good hydrophobicity and biocompatibility, which could greatly compensate the disadvantage of PVA. Moreover, according to the hydrophilic/hydrophobic theory, hydrophilic groups of PVA and P(MAA) are useful for drug release [27]. It is difficult to prepare a PVA/P(MMA-MAA) film using a PVA/P(MMA-MAA) solution; moreover, our aim is to make a tunable hydrophobic–hydrophilic blend film with improved surface characteristics such as a large surface area, globules on film surface, controlled water resistance properties, etc., to make the film useful for various medical applications (drug delivery, wound dressing, etc.). To achieve this goal, we prepared a PVA/P(MMA-MAA) blend film from a PVAc/PMMA blend film through the heterogeneous saponification method and the morphological, thermal, mechanical, and crystallinity properties were explored.

## 2. Materials and Methods

### 2.1. Materials

The MMA and vinyl acetate obtained from Aldrich (St. Louis, MO, USA) were successively washed with NaHSO_3_ aqueous solution and water and were, thereafter, dried using anhydrous CaCl_2_. Subsequently, they were distilled in a nitrogen atmosphere under reduced pressure [28]. The monomer-soluble initiator 2,2′-azobis (2,4-dimethylvaleronitrile) (ADMAN), which was purchased from Wako (Richmond, VA, USA), was recrystallized twice in methanol before being used. PVA, which has a number-averaged molecular weight of 127,000 and a degree of saponification (DS) of 88% (Aldrich Co., St. Louis, MO, USA), was used as a suspension agent. Deionized water was used in all experiments.

### 2.2. Polymerization of PVAc and PMMA

To synthesize the PVAc and PMMA, the suspension polymerization of these monomers was separately performed in a reactor (Duran Co., Mainz, Germany). To synthesize PVAc, the PVA suspension agent was dissolved in water with constant stirring under a nitrogen atmosphere in a 250-mL reactor equipped with a condenser. After the degassing process was completed, the VAc monomer and ADMAN initiator were added to this mixture at 15 °C polymerization temperature, which was subsequently increased to 60 °C. After a 4 h passed, the reaction was terminated, and the resulting mixture was kept for 1 h to isolate the prepared spherical PVAc particles. Subsequently, the reaction mixture was cooled and left for 1 day for precipitation and partition of the PVAc. After this time, the collected PVAc was washed with warm water. The PMMA was also obtained using a similar method.

Equations (1)–(3), which were presented by B. Alhamad et al. [29], were used to calculate the conversion of the individual monomers (VAc and MMA) and overall conversion of polymerization.

d*N^fed^*/d*t* = F (1)

where *N^fed^* is the number of moles fed for the monomer and *F* is the molar flow rate of the monomer.

*X* = 1 − *N*/*N^fed^* (2)

*S* = *XM*/*M* (3)

where *M* is the molecular weight of the monomer, *X* is the conversion of the monomers, and *S* is the overall conversion. The detailed polymerization conditions are listed in Table 1.

### 2.3. Preparation of PVAc/PMMA Blends Film and Its Saponification

The PVAc/PMMA film was prepared using the solution-casting technique. The polymer solutions were obtained by dissolving each polymer in chloroform. The solution concentration was expressed in terms of percentage weight per weight (%*w/w*). The polymer blend solutions were obtained by mixing the polymer solutions. The films were then obtained by casting the solution on glass molds and through successive evaporation of chloroform in a vacuum oven for 5 days at room temperature (25 °C). The dry films were reserved in a plastic bag for the next experiment. The molecular weights of the polymerized PVAc and PMMA are listed in Table 2.

To convert the PVAc/PMMA films into PVA/P(MMA-MAA) films, heterogeneous saponification was carried out in a flask that was simultaneously outfitted with a thermocouple, a dropping funnel, a reflux condenser, and a stirring device. The alkaline solution for the saponification method was obtained by adding NaOH (10 g), Na_2_SO_4_ (10 g), and MeOH (10 g) to the deionized water (100 g), and the PVAc/PMMA film was introduced into the alkali solution at 50 °C temperature. The saponification was maintained and completed after the required time, and PVA and P(MAA) was formed on the surface of the PVAc/PMMA film due to the hydrolysis of both acetate groups in PVAc and the ester group in PMMA in alkali solution. The mixture was then transferred into cold water, and the PVA/P(MMA-MAA) film was set aside for 1 min to precipitate. Finally, the saponified film was dried out for 24 h in a vacuum oven at room temperature after washing with water twice. The thickness and weights of the prepared PVAc/PMMA and treated PVA/P(MMA-MAA) films are listed in Table 3 and Table 4, respectively.

### 2.4. Characterizations

The molecular weights of PMMA and PVAc were calculated using Equation (4) [30]:

[η] = 5.5 × 10^−5^ [M_n_]^0.76^ (in benzene at 25 °C) (4)

where [η] is the intrinsic viscosity. The number-average degree of polymerization (*P_n_*) of PMMA and PVAc was calculated using M_n_. Measurement of the film thickness was performed using a thickness gauge (Ip 65, Mitutoyo, Tokyo, Japan). To measure the solution viscosity, a viscometer (A&D company, Limited, SV-10, Tokyo, Japan) was also used. The surface morphology of the saponified PVA/P(MMA-MAA) film was observed using an optical microscope (Olympus, CKX41, Tokyo, Japan) and atomic force microscope (AFM) (AFM, Park Systems (NX20), Mannheim, Germany). To establish the DS of the saponified PVA/P(MMA-MAA) film, the ratio of the methyl and methylene proton peaks was calculated using a proton nuclear magnetic resonance spectrometer (AVANCE III 500, Bruker, Billerica, MA, USA). To determine the conversion of the PVAc/PMMA film into a PVA/P(MMA-MAA) film, an X-ray diffractometer (XRD) (D/Max-2500, Rigaku, Tokyo, Japan) and an infrared (IR) spectrometer (Frontier, Perkin Elmer, Waltham, MA, USA) were used. The glass transition temperature (T_g_) of the pure PVAc film, PVAc/PMMA blend film, and saponified PVA/P(MMA-MAA) blend film were calculated using a differential scanning calorimetry (DSC Q2000, TA Instruments, and New Castle, DE, USA). The dynamic mechanical analysis (DMA) for studied films were analyzed by using a dynamic mechanical analyzer.

## 3. Results and Discussion

**Figure 1:**
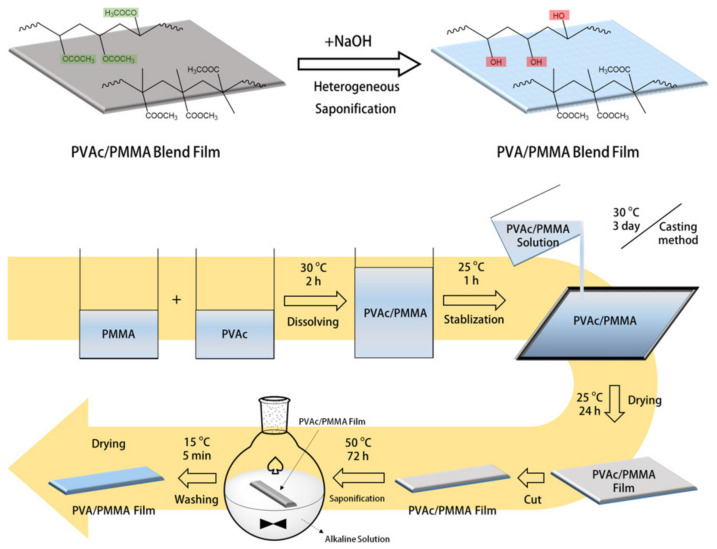
Schematic illustration of the PVA/P(MMA-MAA) blend film prepared using heterogeneous saponification and preparation of the PVAc/PMMA blend film.

Figure 1 shows a schematic of the preparation process of the PVA/P(MMA-MAA) blend film from a PVAc/PMMA blend film using heterogeneous saponification.

### 3.1. XRD Analysis

**Figure 2:**
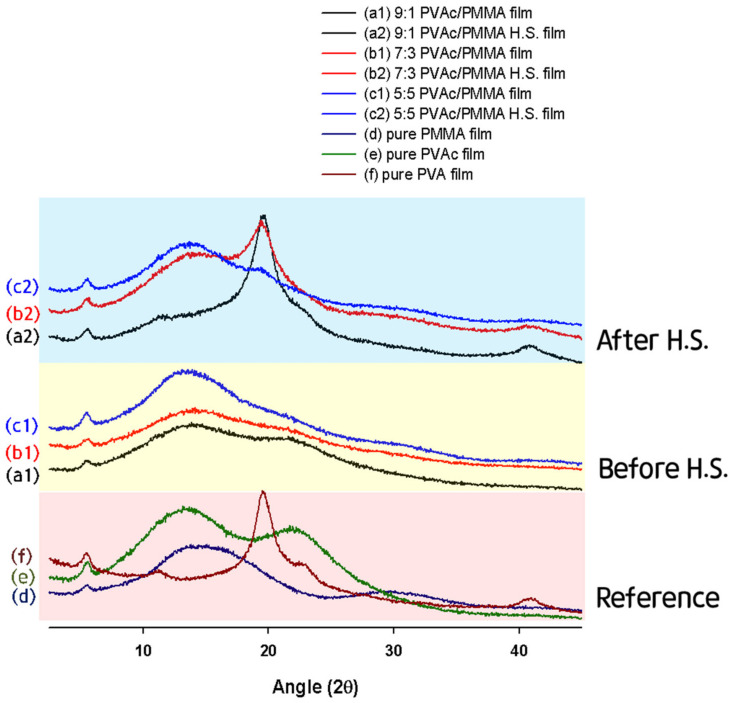
XRD patterns of untreated films (a1, b1, c1, d–f) and treated films (a2, b2, c2).

To detect the structural changes between the untreated and treated films, XRD patterns are obtained. Figure 2 shows the diffractogram of the pure PMMA, PVAc, and PVA films as well as the untreated PVAc/PMMA (9:1, 7:3, 5:5) and treated PVAc/PMMA (9:1, 7:3, 5:5) films prepared by heterogeneous saponification at 50 °C for 72 h. Figure 2 (f) shows that the pure PVA film exhibits three typical diffraction peaks at 2θ = 13.5° (100 lattice plane), 19.8° (101 lattice plane), and 22.5° (200 lattice plane). These three peaks are related to the crystalline regions in the PVA structure [31,32]. We can see that the diffraction pattern of the pure PMMA film exhibits a broad diffraction peak at 2θ = 14°, which is a characteristic of an amorphous material, along with two bands of lower intensities located at 29.7° and 41.7° (Figure 2 (d)) [33]. Additionally, the two broad peaks centered at 15.07° and 22.5° clearly show the amorphous nature of the pure PVAc film (Figure 2 (e)) [34]. In contrast, in the case of the PVAc/PMMA blend film, the typical PVAc peak at 22.5° gradually disappears with the increase in the PMMA content (Figure 2 (a1–c1)). These results indicate the successful formation of PVAc/PMMA blend films with different mass ratios (9:1, 7:3, and 5:5). On the other hand, the treated PVAc/PMMA films with mass ratios of 9:1 and 7:3 (Figure 2 (a2, b2)) exhibit a sharp crystalline peak at 2θ = 19.8°. However, in the 5:5 mass ratio case (Figure 2 (c2)), the peak intensity is comparatively lower because of the higher PMMA content compared with that in the other ratios (9:1 and 7:3). This also proved that the slow saponification of PMMA that occurred compared to PVAc within the designated time may be due to the difference in the saponification reaction mechanism between them, as the functional group of PVAc and PMMA is different. Interestingly, this observed crystalline peak (2θ = 19.8°) matches the diffraction peak of OH groups along the main chain of PVA and P(MMA-MAA) [35,36].

This result validates the successful conversion of PVAc/PMMA film into PVA/P(MMA-MAA) film. Consequently, we can state that saponified PVA/P(MMA-MAA) films can be obtained from PVAc/PMMA films using the heterogeneous saponification method.

### 3.2. TGA Data

**Figure 3:**
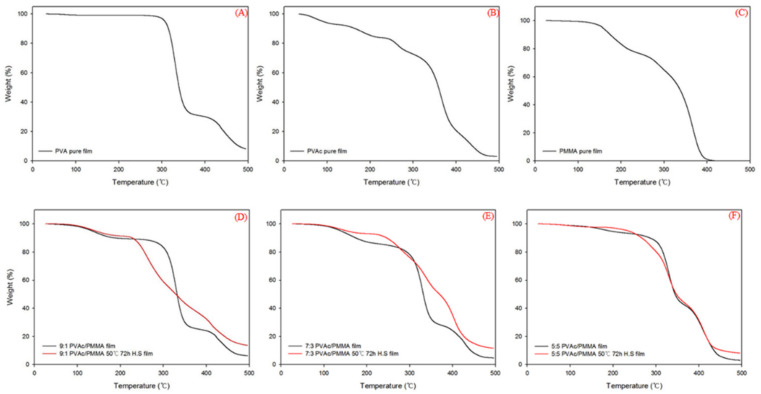
TGA data obtained from (**A**) PVA, (**B**) PVAc, (**C**) PMMA, (**D**) unsaponified and saponified 9:1 PVAc/PMMA blend films, (**E**) unsaponified and saponified 7:3 PVAc/PMMA blend films, and (**F**) unsaponified and saponified 5:5 PVAc/PMMA blend films.

Thermogravimetric analysis (TGA) was performed to explore the thermal stability difference between the untreated and treated films as well as the pure and blend films. Figure 3 shows a typical TGA thermograph of the weight loss as a function of temperature of the pure PMMA, PVAc, and PVA films as well as the untreated PVAc/PMMA (9:1, 7:3, and 5:5) and treated PVAc/PMMA (9:1, 7:3, and 5:5) films prepared using the heterogeneous saponification method at 50 °C for 72 h. Table 5 gives the brief for Figure 3 in thermal parameters of T_onset_, T_10%_, T_50%,_ T_max_, and residue. From Figure 3, it was seen that each of the saponified and unsaponified blend films show only one T_onset_ as observed on their thermograms. This indicates the presence of a chemical interaction between PVAc and PMMA [37], and hydrogen bonding interactions between PVA and P(MMA-MAA) in each unsaponified and saponified blend film, respectively.

**Figure 4:**
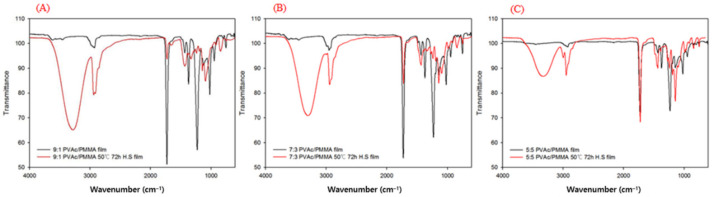
FTIR patterns of (**A**) unsaponified and saponified 9:1 PVAc/PMMA blend films, (**B**) unsaponified and saponified 7:3 PVAc/PMMA blend films, and (**C**) unsaponified and saponified 5:5 PVAc/PMMA blend films.

Overall, major weight losses were observed in the range from 200–500 °C for all types of films, which may have been related to the structural disintegration of the polymers and volatilization of the polymer yields [38,39,40,41,42]. Figure 3 shows that both untreated PVAc/PMMA (9:1, 7:3, and 5:5) and treated PVAc/PMMA (9:1, 7:3, and 5:5) films exhibit higher thermal stability than the pure PVAc and PMMA films during this period (Figure 3B–F). This result can be attributed to the chemical interaction between PVAc and PMMA [43] and PVA and P(MMA-MAA). Evidently, all types of treated films exhibited comparatively lower weight loss than the untreated films (Figure 3D–F), possibly due to the conversion of PVAc/PMMA into PVA/P(MMA-MAA) (Figure 2 and Figure 4). Moreover, both treated and untreated PVAc/PMMA (5:5) films exhibited higher thermal stability (T10% was 285.91 °C and 266.96 °C, respectively, as shown in Table 5) than the other two blend films (Figure 3D–F) up to 300 °C. The increased PMMA content in PVAc improved thermal stability and may be due to its barrier properties that cause the delay of oxygen permeation and the discharge of volatile products after degradation [44].

Moreover, T_50%_, i.e., decomposition temperature of 50% material, occurred at 350.13, 390.12, and 338.72 °C for general PVA, unsaponified pure PVAc film and unsaponified pure PMMA film, and among all treated and untreated blend film the highest T_50%_ value (369.8 °C) was obtained for saponified 7:3 PVAc/P(MMA-MAA) film. The reason for the lower T50% value of saponified 9:1 PVAc/P(MMA-MAA) film compared to saponified 7:3 PVAc/P(MMA-MAA) film may be due to an inherently lower T_50%_ value (350.13 °C) of general PVA than PVAc (390.12 °C) as the saponified 9:1 ratio had more PVA than the saponified 7:3 ratio and strong H bonds may have occurred between two –OH groups as sufficient MAA content existed at the saponified 7:3 ratio [44]. However, the T_50%_ value again decreased for the saponified 5:5 ratio compared to saponified 7:3 and may be due to a weak PVA and P(MMA-MAA) interaction at this blend ratio after saponification.

The residual mass gradually decreased with the PMMA content for both treated and untreated film, as shown in Table 5, because the residual mass of PMMA is the lowest of all the films investigated. Finally, improved thermally stable PVA/P(MMA-MAA) (9:1, 7:3, and 5:5) films can be obtained from the PVAc/PMMA (9:1, 7:3, and 5:5) films using the heterogeneous saponification method.

### 3.3. FTIR Analysis

A Fourier transform IR (FTIR) analysis was conducted to illustrate the effects of heterogeneous saponification on the PVAc/PMMA film. The FTIR patterns of the unsaponified and saponified PVAc/PMMA films with different mass ratios of 9:1, 7:3, and 5:5 is shown in Figure 4. In the case of the unsaponified (PVAc/PMMA) (9:1, 7:3, and 5:5) films, the vibrational bands centered at 2923 and 2865 cm^−1^ were related to the CH_3_ asymmetric and symmetric stretching of PVAc, respectively (Figure 4A–C) [45]. Additionally, some of the significant peaks attributed to the PMMA structures were located at the following: from 2899 to 2950 cm^−1^ (CH_2_ stretching vibration); 1274 cm^−1^ (C–O stretching vibration); 1195 cm^−1^ (-O–CH_3_ stretching vibration); and 1144 cm^−1^ (CH_3_ stretching) (Figure 4A–C) [46]. We also observed that the intensity of the peaks varied depending on the mass ratio of PVAc and PMMA (Figure 4A–C). On the other hand, after the saponification process, the major changes observed in all types of films (PVAc/PMMA) (9:1, 7:3, and 5:5) were the broad bands that appear in the 3600–3200 cm^−1^ region due to the O–H stretching generated from both intermolecular and intramolecular hydrogen bonds [47]. Moreover, the intensity of the peak located from 2899 to 2950 cm^−1^ due to the CH_2_ stretching vibration increased. These observed changes in the vibration bands were due to the conversion of PVAc/PMMA to PVA/P(MMA-MAA). The other minor changes in the peak intensity in the 500–2000 cm^−1^ region was also due to the formation of PVA/P(MMA-MAA) from PVAc/PMMA. Figure 4A–C shows the peak intensity also varied depending on the mass ratios of PVAc and PMMA and the O–H peak for saponified film containing more PMMA, which shows lower intensity due to the difference in the saponification reaction rate between PVAc and PMMA at the designed time, which also increased the PMMA content and affected the saponification rate of PVAc.

### 3.4. DMA Analysis

**Figure 5:**
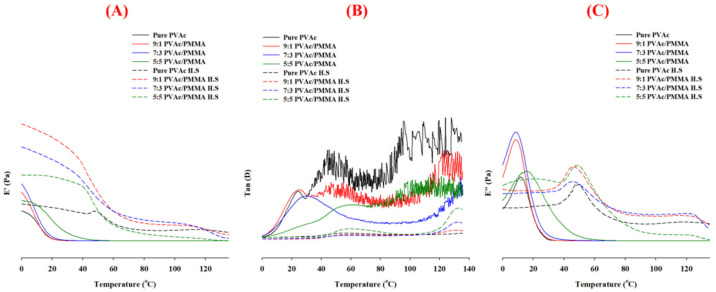
DMA analysis: (**A**) Storage modulus (E′), (**B**) loss factor (tan D); and (**C**) loss modulus (E″), of treated and untreated PVAc, and PVAc/PMMA blend films with different mass ratios of 9:1, 7:3, and 5:5.

Generally, the miscible polymer blend showed only one glass transition temperature (T_g_) obtained by the homogeneous state of the amorphous region, whereas immiscible blends had two T_g_s, one for each individual component. Figure 5 displays the dynamic mechanical analysis (DMA) of treated and untreated PVAc and PVAc/PMMA blend films with different mass ratios of 9:1, 7:3, and 5:5.

Figure 5 demonstrates the variations of the storage modulus (E′) of treated and untreated neat PVAc and PVAc/PMMA blend films as a function of temperature. Firstly, the E′ of all untreated neat PVAc and PVAc/PMMA blend film dropped sharply in the temperature range from 10–50 °C, because of their glass transitions whereas all treated films also showed a single relaxation phase in the investigated temperature range (0–150). Second, when the temperature was above approximately 45 °C, adding PMMA to the PVAc/PMMA blends had no influence on the E′ because both components were in a glass state. The increase in the E′ with the PMMA content of PVAc/PMMA blends was attributed to the reinforcement of the PMMA component due to its high stiffness in the glassy state. The storage modulus of the treated PVAc film was higher than that of the untreated PVAc film, which increased with increasing quantities of PMMA in the treated PVAc/PMMA film, with the exception of the 5:5 ratio, which is attributed to the difference in polymer chain mobility [48] and which occurred due to the difference in the conversion rate and interaction strength between PVA and P(MMA-MAA) after saponification, as evidenced by XRD and FTIR results (Figure 2 and Figure 4).

The T_g_, which is a measure of polymer chain mobility, can be calculated using the tan peak δ, and is summarized in Table 1. The glass transition of neat PVAc was seen as a strong single tan δ peak at roughly 24.1 °C. In the case of all unsaponified and saponified (PVAc/PMMA) (9:1, 7:3, and 5:5), the blend films show only one peak in the tan δ spectrum (Figure 2). These results suggest that a single T_g_ peak represents a unique phase in the prepared polymeric films [49]. Notably, it could be observed that the T_g_ of PVAc component shifted to higher temperatures with increasing PMMA content. Such a change of T_g_ with an increase of PMMA content indicated that there was an intercompositional interaction between PVAc and PMMA in the amorphous region, which increased the compatibilization in the PVAc/PMMA film [50]. All the saponified films had a larger T_g_ than the unsaponified films, which is supported by the E′ result, and the unsaponified films had a similar tendency as the unsaponified films, i.e., there was also an interaction between PVA and P(MMA-MAA) in the PVA/P(MMA-MAA) film. Unsaponified (PVAc/PMMA) (9:1, 7:3, and 5:5) blend films had T_g_ values of 24.1, 25.5, 30.0, and 59.1 °C, respectively, while saponified (PVAc/PMMA) (9:1, 7:3, and 5:5) blend films had T_g_ values of 56.6, 58.0, 58.2, and 59.6 °C, respectively. These results can be explained by the high miscibility between PVAc and PMMA, and PVA and P(MMA-MAA) confirmed by the presence of a single T_g_ peak in the blend films and the results (T_g_) are consistent with previous reports [51,52,53].

The changes of the loss modulus (E″) versus the temperature curve for treated and untreated neat PVAc and PVAc/PMMA blend films, as a function of temperature, are also displayed in Figure 5, and peaks correspond to the glass transition [54]. T_g_ values determined by E″ and tan D curves are shown in Table 6, and the difference in tendency noticed for T_g_ obtained by both curves may be due to the temperature difference of the peak location because when the temperature rises, the viscosity of the polymer decreases gradually [55]; however, for most of the cases, an acceptable difference (up to 25 °C) in the temperature range was observed [54]. According to the E″ curve, for the unsaponified film, the T_g_ value decreased with the increase in PMMA, however, again, an increase for the 5:5 ratio indicates that more PMMA is necessary to increase the T_g_ of the PVAc matrix. This indicates that interactions between two polymers occurred, resulting in mutual solubility and a change in T_g_ at the same time. On the other hand, saponified films showed higher T_g_ than unsaponified films, consistent with the E′ results and their similar tendencies. However, the addition of PMMA slightly decreased the T_g_ of PVAc for all blend ratios and may be due to the conversion of PMMA to P(MMA-MAA) in which more hydrogen bonding occurred between the hydroxyl group, which is responsible for the decrease in T_g_ by increasing the elasticity of the polymer matrix [56].

### 3.5. DSC Analysis

**Figure 6:**
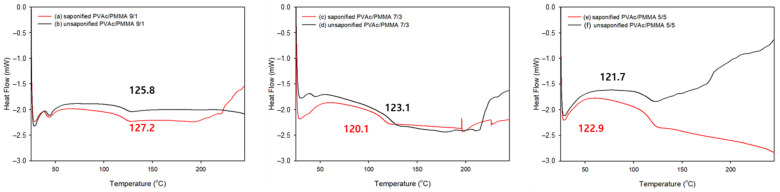
DSC data of (**a**) saponified and (**b**) unsaponified 9:1 PVAc/PMMA blend films, (**c**) saponified and (**d**) unsaponified 7:3 PVAc/PMMA blend films, and (**e**) saponified and (**f**) unsaponified 5:5 PVAc/PMMA blend films.

A DSC analysis was performed on the alkaline treated and untreated PVAc/PMMA blend films with different mass ratios of 9:1, 7:3, and 5:5 (Figure 6). T_g_ of the PVAc/PMMA 9/1 film exhibited an endothermic peak at 125.8 °C for the untreated and at 127.2 °C for the alkali treated films (Figure 6 (a)). However, the enthalpy-change value was similar. The endothermic peak shifted to a slightly higher temperature compared with that in the untreated films, which was from 125.8 to 127.2 °C. T_g_ of the untreated PVAc/PMMA agrees well with the other reported results [57,58]. Similar results are obtained in the case of the other blend films (PVAc/PMMA 5:5), which demonstrate a higher thermal property (T_g_) after the saponification treatment, which could be due to the conversion of PVAc/PMMA into PVA/P(MMA-MAA) blend films because PVA possesses a higher thermal property (Tg) than PVAc [59]. However, saponified PVAc/PMMA 7:3 film showed a lower thermal property (T_g_) than the unsaponified PVAc/PMMA 7:3 film, which could be due to a weak PVA and P(MMA-MAA) interaction after saponification, as higher conversion of PMMA to P(MMA-MAA) occurred at this blend ratio after saponification than at other ratios (9:1 and 5:5) (Figure 2). It is also observed that both saponified and unsaponified PVAc/PMMA 9/1 film showed higher T_g_ than other studied films because of the higher degree of saponification (Figure 6), i.e., higher conversion of PVAc to PVA and probable strong chemical interaction at this blend ratio. According to the figure, the absence of melting temperature (T_m_) and crystalline temperature (T_c_) for all treated and untreated blend films can be attributed to the amorphous nature of both PVAc and PMMA [57]. The glass transition temperature values of DMA and DSC were different, however, within the expected range [57].

**Figure 7:**
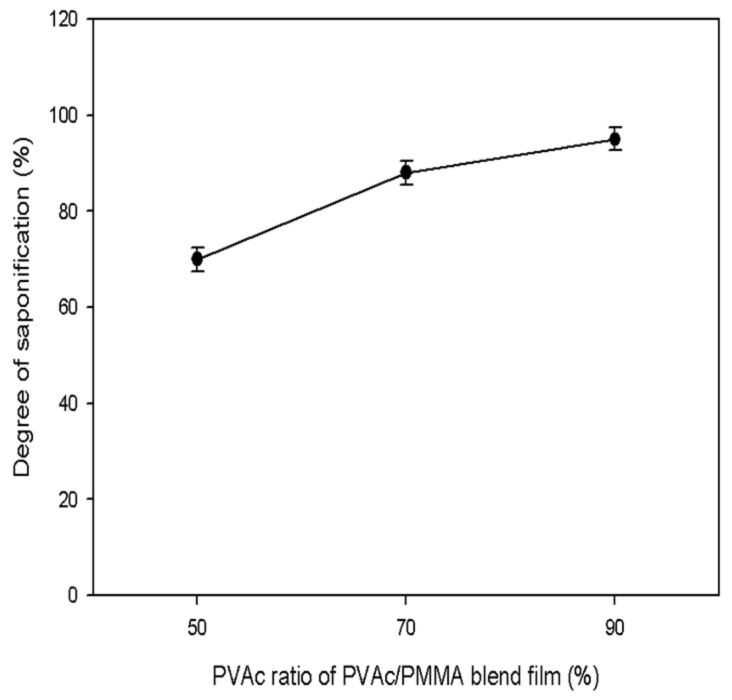
Effect of PVAc content on DS of the PVAc/PMMA blend film.

The effects of the PVAc content on the DS of the PVAc/PMMA film are shown in Figure 7. The obtained DS value clearly increased with the increase in the PVAc content in the PVAc/PMMA blend film. Most likely, the higher content of PMMA slowed the saponification process, thus, the higher DS value obtained at the higher PVAc ratio, indicates a lower PMMA content.

### 3.6. Morphological Analysis

**Figure 8:**
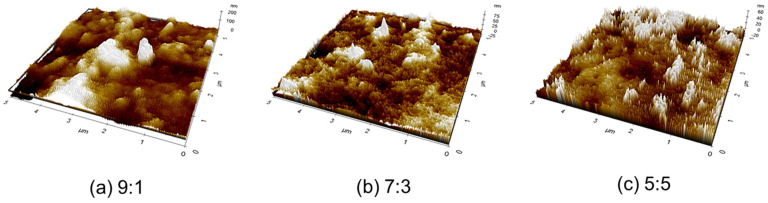
AFM images of (**a**) saponified 9:1 PVAc/PMMA, (**b**) saponified 7:3 PVAc/PMMA, and (**c**) saponified 5:5 PVAc/PMMA blend films.

The surface morphology of the saponified PVAc/PMMA blend film with different mass ratios of 9:1, 7:3, and 5:5 was investigated using an atomic force microscope. The results are shown in Figure 8. A comparison of Figure 8a–c reveals that a definite morphological change occurred with the decrease in the PVAc content in the PVAc/PMMA blend. The height histogram of the surface morphology of the PVAc/PMMA blend film reflects the significant roughness of the surface feature. The surface roughness of the saponified PVAc/PMMA blend film with different mass ratios of 9:1, 7:3, and 5:5 was 78, 54, and 46 nm, respectively. This result confirms that the morphological variation among the saponified PVAc/PMMA blend films with different mass ratios could have most likely influenced the various PVAc content in the DS of the PVAc/PMMA blend films (Figure 7).

**Figure 9:**
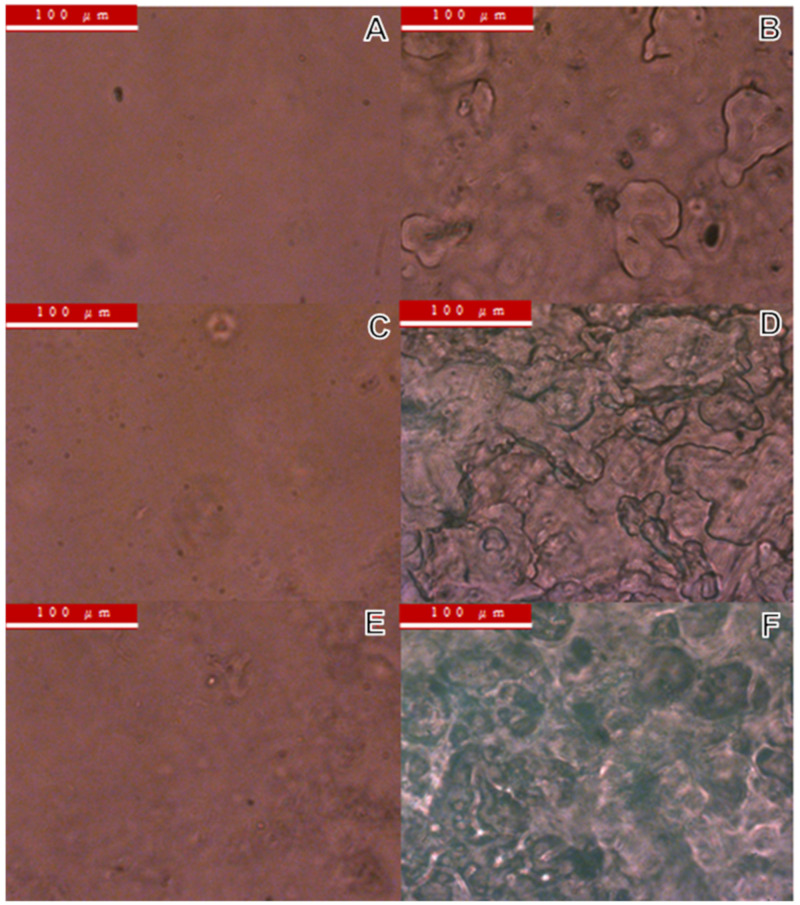
OM images of the (**A**) unsaponified and (**B**) saponified 9:1 PVAc/PMMA blend films, (**C**) unsaponified and (**D**) saponified 7:3 PVAc/PMMA blend films, and (**E**) unsaponified and (**F**) saponified 5:5 PVAc/PMMA blend films.

Figure 9 shows the optical images taken at the same magnification that indicate the unsaponified and saponified PVAc/PMMA blend films with different mass ratios of 9:1, 5:5, and 7:3. As expected, we observed that the surface roughness (globules, void, and hollow) of all types of films increase after the saponification (Figure 9B,D,F). Consequently, the surface area also increased after the saponification. The possible reasons for the change in the film surface after saponification could be the increase in the film density and PVA skins that are dissolved in the aqueous saponification solution. In addition, the PVAc molecule is composed of a chain of PVA molecules; thus, reaction with strong alkali occurred. During the violent reaction, the organization of the polymer chain may have changed, or the chain end became small [20]. We also noticed that the film surface became rougher when the PMMA mass ratio in the PVAc/PMMA blend film increased (Figure 9A–F), possibly because the continuity of the PVAc was hindered by the increase in the PMMA content.

## 4. Conclusions

In this study, a blend film of hydrophilic PVA with hydrophobic–hydrophilic P(MMA-MAA) with different mass ratios of 9:1, 7:3, and 5:5 was prepared using heterogeneous saponification of PVAc/PMMA with different mass ratios of 9:1, 7:3, and 5:5, respectively. According to the AFM results, morphological variations among the saponified PVAc/PMMA blend films with different mass ratios of 9:1, 7:3, and 5:5 were observed. The OM results revealed that the saponified PVAc/PMMA films demonstrated increased surface roughness and area compared with the unsaponified PVA/P(MMA-MAA) films. We also observed that the DS value was dependent on the PVAc content. The TGA and DSC results confirmed that improved thermally stable saponified PVA/P(MMA-MAA) (9:1, 7:3, and 5:5) films were obtained. Finally, the XRD and FTIR results provided evidence of the conversion of PVAc/PMMA films into a PVA/P(MMA-MAA) film. Overall, this work could expand the applications of PVA/P(MMA-MAA) in medical materials and offer a novel idea for preparing a hydrophilic/hydrophobic–hydrophilic blend film with improved surface properties for medical applications through the heterogeneous saponification system.

## Figures and Tables

**Table 1:** Conditions of the suspension polymerization of VAc and MMA.

Condition	Value	Value
Type of initiator	ADMVN	ADMVN
Type of suspending agent	PVA	PVA
Initiator concentration	0.001 mol/mol of VAc	0.001 mol/mol of MMA
Suspending agent concentration	1.5 g/dL of water	1.5 g/dL of water
Monomer/water	0.5 L/L VAc/L	0.5 L/L MMA/L
Rpm	300	300
Temperature	60 °C	60 °C

**Table 2:** Molecular weights of the polymerized PVAc and PMMA.

Sample	M_w_ (g/mol)	M_n_ (g/mol)	Polydispersity Index (M_w_/M_n_)
PVAc	495,266	201,851	2.45
PMMA	756,433	249,700	3.02

**Table 3:** Thickness of the prepared films.

Before (µm)		After (µm)	
PMMA	187 ± 15	PMMA	186 ± 12
PVAc	184 ± 15	PVAc	168 ± 13
PVAc/PMMA (9:1)	183 ± 12	PVAc/PMMA (9:1)	170 ± 11
PVAc/PMMA (7:3)	184 ± 11	PVAc/PMMA (7:3)	173 ± 11
PVAc/PMMA (5:5)	185 ± 10	PVAc/PMMA (5:5)	177 ± 12

**Table 4:** Weight of the prepared and treated films.

Before (g)		After (g)	
PMMA	0.542 ± 15	H.S. PMMA	0.532 ± 12
PVAc	0.484 ± 11	H.S. PVAc	0.305 ± 11
PVAc/PMMA (9:1)	0.461 ± 12	H.S. PVAc/PMMA (9:1)	0.291 ± 11
PVAc/PMMA (7:3)	0.453 ± 12	H.S. PVAc/PMMA (7:3)	0.309 ± 14
PVAc/PMMA 5:5)	0.473 ± 11	H.S. PVAc/PMMA (5:5)	0.346 ± 15

**Table 5:** TGA data of the studied films measured at a heating rate of 10 °C/min.

Sample Name	T_onset_ (°C)	T_5%_ (°C)	T_50%_ (°C)	T_max_ (°C)	Residue
General PVA	330.11	340.22	350.13	375.27	10.02
Pure PVAc	350.22	195.14	390.12	475.12	4.95
Pure PMMA	275.75	175.07	338.72	395.55	0.04
9:1 PVAc/PMMA	292.31	187.91	332.18	351.45	6.20
9:1 PVAc/PMMA H.S	224.44	227.94	329.22	397.27	13.60
7:3 PVAc/PMMA	299.43	174.49	331.92	354.04	4.65
7:3 PVAc/PMMA H.S	244.76	246.43	369.68	424.60	11.57
5:5 PVAc/PMMA	300.34	285.91	344.55	442.28	3.05
5:5 PVAc/PMMA H.S	229.73	266.96	347.63	429.30	8.12

**Table 6:** Comparison of Tg values determined by peaks in tan δ and E″ curves for all prepared and treated PVAc/PMMA blend film.

Blend	Tan δ (°C)	E″ (°C)
Pure PVAc	24.1	11.9
9:1 PVAc/PMMA	25.5	8.6
7:3 PVAc/PMMA	30	8.5
5:5 PVAc/PMMA	59.1	15.9
Pure PVAc H.S film	56.6	49
9:1 PVAc/PMMA H.S film	58	46.5
7:3 PVAc/PMMA H.S film	58.2	45.9
5:5 PVAc/PMMA H.S film	59.6	48.4

## Data Availability

Not applicable.
